# Real-world study of SHR-1210 plus apatinib in the treatment of BRAF-negative mucosal melanoma: efficacy, safety and implications of precision medicine

**DOI:** 10.3389/fimmu.2026.1755669

**Published:** 2026-04-21

**Authors:** Yong Zhang, Ting Wang, An-Qi Lyu, Fang Zhang, Fang-Hui Li, Ling-Di Zhao, Zi-Bing Wang, Yi-Man Shang, Bao-Zhen Ma, Xiao-Qun Dong, Quan-Li Gao

**Affiliations:** 1 Department of Immunotherapy, The Affiliated Cancer Hospital of Zhengzhou University & Henan Cancer Hospital, Zhengzhou, China; 2 Department of Radiation Oncology, The Affiliated Cancer Hospital of Zhengzhou University & Henan Cancer Hospital, Zhengzhou, China; 3 Precision Health Program, Department of Radiology, College of Human Medicine, Michigan State University, East Lansing, MI, United States

**Keywords:** anti-PD-1 antibody, apatinib, immunotherapy, mucosal melanomas (MM), SHR-1210

## Abstract

**Background:**

Mucosal melanomas arise from melanocytes in mucosal tissues, and it’s usually detected at a late stage owing to its anatomic location. Advances in immunotherapies have proved to be promising therapeutic approaches for advanced mucosal melanoma patients, however, clinical studies have shown mucosal melanoma patients are less responsive to ICIs than cutaneous melanoma. Effective therapy is still lacking.

**Objective:**

To investigate the efficacy and safety of SHR-1210 (an anti-programmed cell death-1 antibody) in combination with Apatinib (a vascular endothelial growth factor receptor 2 inhibitor) as late-line treatment in patients with advanced mucosal melanoma (Ethics approval number 2019184;ClinicalTrials.gov ID: NCT03986515).

**Patients and methods:**

Patients with confirmed metastatic mucosal melanoma according to AJCC 8.0. In this single-arm, single-center, phase II nonrandomized clinical trial, patients with advanced mucosal melanoma who have received several line treatments were enrolled between 2019.06 and 2022.12. The data cutoff date was June 11th, 2025. Patients received 200mg SHR-1210 intravenously every 3 weeks, and oral Apatinib 250 mg daily until unacceptable toxic effects or disease progression.

**Results:**

A total of 13 patients were enrolled and received treatment. The disease control rate (DCR) reached 100% (SD = 13). The objective response rate (ORR) was 0%. The estimated median overall survival (OS) was 29.9 months (95% CI 15.26-44.54),. The estimated median progression-free survival (PFS) was 5.17 months (95% CI 3.27-10.27),. The most common grade 1–2 was hand and foot induration with desquamation and pain, and one case of grade 4 abnormal liver function and grade 3 hypertension. No treatment-related deaths occurred.

**Conclusions:**

This suggests that this combination therapy model may be a promising disease control for mucosal melanomas. However, the sample size is relatively small and needs to be increased for further research.

**Clinical Trial Registration:**

https://clinicaltrials.gov/study/NCT03986515, identifier NCT03986515.

## 1 Introduction

Mucosal melanoma (MM) is a rare and aggressive subtype of melanoma arising from melanocytes in mucosal surfaces of the respiratory, gastrointestinal, and genitourinary tracts (1). MM is notably more prevalent in Asian populations. Its diagnosis is frequently delayed because lesions in these concealed anatomical sites lack early visible signs, unlike cutaneous melanoma (CM) (2). Compared with cutaneous melanoma, MM is biologically distinct, characterized by a lower tumor mutational burden, infrequent BRAF V600 mutations, and enrichment of KIT or NRAS alterations (3). These features contribute to its limited responsiveness to standard systemic therapies and to its poor prognosis, with five-year survival rates for metastatic disease of less than 20% (2).

Immune checkpoint inhibitors (ICIs) have transformed the management of cutaneous melanoma, yet their benefits in MM are modest. *Post-hoc* analyses of pivotal trials with PD-1 inhibitors showed objective response rates (ORRs) of only 10–20% in MM, compared with over 30% in cutaneous melanoma (4, 5). Even dual blockade with PD-1 and CTLA-4 has yielded limited improvements (6). Thus, effective systemic treatment strategies for advanced MM remain an urgent unmet need.

Angiogenesis plays a central role in MM biology. Vascular endothelial growth factor (VEGF) not only promotes neovascularization but also fosters an immunosuppressive tumor microenvironment (7). Preclinical studies have shown that simultaneous VEGF and PD-1 blockade enhances T-cell infiltration and antitumor immunity (8). Early-phase trials combining PD-1 antibodies with VEGF receptor tyrosine kinase inhibitors (VEGFR-TKIs) such as axitinib or Apatinib have reported ORRs of 24–48% and median progression-free survival (PFS) of 7–8 months, with manageable toxicity profiles (9–11).

Despite these promising results, most evidence derives from clinical trial populations with limited generalisability. Real-world data are scarce, particularly for patients with heavily pretreated, BRAF-negative MM. SHR-1210 (camrelizumab), a PD-1 monoclonal antibody, combined with Apatinib, a selective VEGFR-2 inhibitor, represents a rational therapeutic strategy to overcome the intrinsic resistance of MM to immunotherapy alone. We therefore conducted a real-world, single-center, phase II study to evaluate the efficacy, safety, and clinical implications of SHR-1210 plus Apatinib in patients with advanced, BRAF-negative MM.

## 2 Patients and methods

### 2.1 Patients and study design

This is a single-arm, open-label, phase II clinical trial conducted at a single center. The trial aims to evaluate the efficacy and safety of SHR-1210, an anti-PD-1 monoclonal antibody, in combination with Apatinib, a VEGFR-2 inhibitor, as a late-line treatment for advanced BRAF-negative mucosal melanoma. Eligible patients were adults aged 18 to 75 years, diagnosed with metastatic mucosal melanoma according to the AJCC 8.0 staging system. Inclusion criteria included at least one measurable lesion per RECIST v1.1 and an Eastern Cooperative Oncology Group (ECOG) performance status of 0–2. All patients had received prior systemic therapy, excluding immunotherapy.

Patients with active autoimmune diseases, uncontrolled infections, or prior treatment with PD-1, PD-L1 inhibitors, or VEGFR inhibitors were excluded. Written informed consent was obtained from all participants prior to enrollment. The study was approved by the ethics committee (approval number 2019184) and registered at ClinicalTrials.gov (NCT03986515).

### 2.2 Treatment and assessment

Patients received SHR-1210 intravenously at a dose of 200 mg every 3 weeks and oral Apatinib at 250 mg daily. Treatment continued until disease progression, unacceptable toxicity, or patient withdrawal. Safety assessments were conducted throughout the study, with adverse events (AEs) graded according to the National Cancer Institute Common Terminology Criteria for Adverse Events (CTCAE v4.03). Patients underwent imaging assessments at baseline, every 6 weeks during the first 12 cycles, and then every 9 weeks thereafter, to evaluate tumor response according to RECIST v1.1 and immune-related RECIST (irRECIST) criteria.

The primary endpoint was overall response rate (ORR), defined as the proportion of patients achieving a complete response (CR) or partial response (PR) based on RECIST v1.1. Secondary endpoints included disease control rate (DCR), progression-free survival (PFS), overall survival (OS), and safety profiles. Biomarker analyses were performed using tumor biopsies to assess tumor mutational burden (TMB), PD-L1 expression, and other genetic alterations.

### 2.3 Statistical analysis

Efficacy analyses included all patients who received at least one dose of treatment and underwent at least one response evaluation. ORR and DCR were calculated with 95% confidence intervals using the Clopper-Pearson method. PFS and OS were assessed using the Kaplan-Meier method, with median values and 95% confidence intervals provided. Subgroup comparisons were performed using the log-rank test. Continuous variables were expressed as median (range), while categorical variables were reported as frequency (percentage). Safety analyses were conducted on all patients who received at least one dose of study medication. Statistical significance was set at P < 0.05, and all analyses were conducted using SPSS v22.0 and GraphPad Prism software.

## 3 Results

### 3.1 Patient characteristics

Between June 2019 and December 2022, a total of 13 patients with BRAF-negative advanced mucosal melanoma were enrolled and treated. As summed in **Table 1**, the median age was 57 years (range, 42–71 years), with 9 patients (69.23%) aged ≥65 years. Male patients accounted for 30.77% (4/13). Primary tumor sites included the nasal cavity (2 patients), rectum (2 patients), esophagus (2 patients), gingiva (2 patients), sinus (2 patients), genital tract (2 patients), and upper lip (1 patient). All patients had stage IV disease according to the AJCC 8th edition, with the majority (69.23%) presenting with M1c metastasis. The most common metastatic sites were lymph nodes (84.62%) and liver (46.15%). Eleven patients (84.62%) had fewer than three metastatic organ sites involved.

**Table 1:** Distribution of patient characteristics (n=13).

Characteristics	Values
Age (years)	
Median (range)	57 (42-71)
<65	4 (30.77%)
≥65	9 (69.23%)
Sex	
Female	9 (69.23%)
Male	4 (30.77%)
Location of primary lesion	
Nasal cavity	2 (15.38%)
Oral cavity	3 (23.08%)
Esophagus	2 (15.38%)
Rectum	2 (15.38%)
Sinus	2 (15.38%)
Genital tract	2 (15.38%)
Metastasis status in stage IV*	
M1a	3 (23.08%)
M1b	1 (7.69%)
M1c	9 (69.23%)
M1d	0
Metastatic site	
Liver	6 (46.15%)
Lung	2 (15.38%)
Bone	0
Lymph nodes	11 (84.62%)
Brain	0
Pancreas	2 (15.38%)
Paranephros	1 (7.69%)
Skin	1 (7.69%)
Parotid gland	1 (7.69%)
Tongue	1 (7.69%)
Breast	1 (7.69%)
Number of metastatic organ sites with disease	
<3	11 (84.62%)
≥3	2 (21.43%)
Number of prior anticancer regimens	
1	10 (76.92%)
2	3 (23.08%)
Mutation status	
CIK	2 (15.38%)
NRAS	1 (7.69%)
NF1	1 (7.69%)
Hras EX2	1 (7.69%)
BRAF/RAS/CKIT/KRAS triple wild type PD-L1 status	5 (38.46%)
Positive	1 (7.69%)
Negative	4 (30.77%)
Not available	8 (61.54%)
Sum of diameters of target lesions (mm)	
<50	7 (53.85%)
≥55	6 (46.15%)
Lactate Dehydrogenase LDH	U/L (109-245)
<245	10 (76.92%)
≥245	3 (23.08%)
Subsequent treatment	
Chemotherapy combined with antiangiogenic therapy	5 (38.46%)
Immunotherapy combined with antiangiogenic therapy	2 (15.38%)
Other ICIs therapy	1 (7.69%)
Chemotherapy combined with immunotherapy	2 (15.38%)
Interferon combined with ICIs therapy	1 (7.69%)
Treatment with traditional Chinese medicine and herbal remedies	1 (7.69%)
Symptomatic treatment	1 (7.69%)
The expression level of VEGFR-2 protein by IHC	
0	9 (69.23%)
+	3 (23.08%)
++	1 (7.69%)
+++	0

* means: Melanoma staging is based on the 8th edition of the American Joint Committee on Cancer (AJCC).

Regarding prior anticancer therapy, 10 patients (76.92%) had received one prior line of systemic treatment, and 3 patients (23.08%) had received two prior lines. No patient had prior exposure to immunotherapy or VEGFR inhibitors. Mutation status analysis showed that 5 patients (38.46%) were triple wild-type for BRAF/RAS/CKIT/KRAS, while NRAS, NF1, and HRAS exon 2 mutations were each identified in one patient (7.69%). PD-L1 expression was positive in one patient (7.69%), negative in four (30.77%), and not available in the remaining eight (61.54%). The sum of target lesion diameters was <50 mm in 7 patients (53.85%). Serum lactate dehydrogenase (LDH) levels were within normal range (<245 U/L) in 10 patients (76.92%).

VEGFR-2 protein expression was assessed by immunohistochemistry (IHC): 9 patients (69.23%) showed no expression (0), 3 patients (23.08%) had weak positivity (+), and 1 patient (7.69%) had moderate positivity (++); no patient exhibited strong positivity (+++).

### 3.2 Clinical outcomes

**Figure 1:**
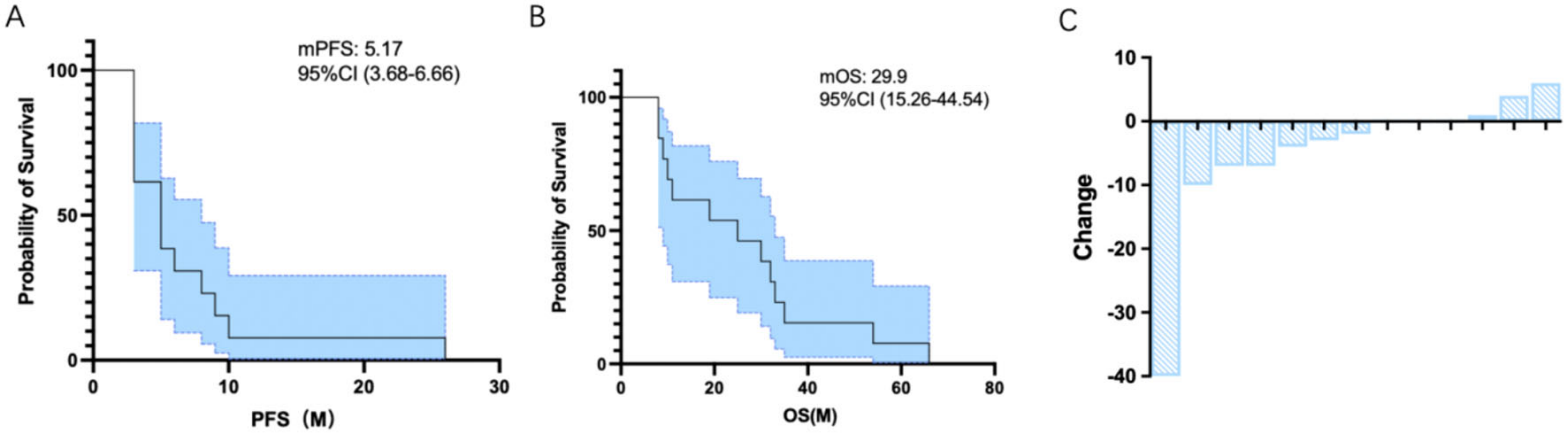
Characteristics of tumor response. **(A)** Kaplan-Meier plot of PFS; **(B)** Kaplan-Meier plot of OS; **(C)** waterfall plot of efficacy.

Among the 13 patients, all patients were evaluated. There is no censored data, and we confirm all patients have experienced the death. As of the data cutoff date (June 11, 2025), the median follow-up duration was 29.9 months. The objective response rate (ORR) was 0%; however, all patients achieved stable disease (SD) as their best response, resulting in a disease control rate (DCR) of 100% (13/13). The median progression-free survival (PFS) was 5.17 months (95% CI, 3.27–10.27) (**Figure 1A**), and the median overall survival (OS) was 29.9 months (95% CI, 15.26–44.54) (**Figure 1B**). Notably, several patients maintained stable disease for more than 12 months, indicating durable benefit in a subset of patients. The waterfall plot of efficacy showed in **Figure 1C**.

### 3.3 Summary of adverse events

6 (6/13) patients experienced treatment-related adverse events (AEs). The most common grade 1–2 AEs were hand–foot syndrome-like dermatologic changes, including hyperkeratosis, desquamation, and pain; some patients also developed mild to moderate fatigue and hypertension. Grade ≥3 AEs included one case of grade 3 hypertension, one case of grade 3 rash with grade 4 hepatic dysfunction, and one case of grade 3 hypoproteinemia. Three patients discontinued treatment due to severe AEs (including pulmonary embolism, hepatic dysfunction, and hypoproteinemia), but no treatment-related deaths occurred. Overall, the toxicities associated with this regimen were manageable and could be mitigated through dose adjustment or supportive care. All this summed in (**Table 2**).

**Table 2:** Treatment-Related adverse events in the SHR-1210 plus apatinib combination study.

All	No. of patients (%)					
Treatment-related adverse events	All Grades	Grade 1	Grade 2	Grade 3	Grade 4	Grade 5
All	6 (42.86)	7 (50)	2 (14.29)	3 (21.43)	1 (7.14)	0
Hand and foot syndrome	3 (21.43)	2 (14.29)	1 (7.14)	0	0	0
Hypertension	2 (14.29)	0	1 (7.14)	1 (7.14)	0	0
Fatigue	2 (14.29)	2 (14.29)	0	0	0	0
hypoproteinemia	1 (7.14)	0	0	1 (7.14)	0	0
Abnormal liver enzymes	1 (7.14)	0	0	0	1 (7.14)	0
Hyponatremia	1 (7.14)	1 (7.14)	0	0	0	0
Pulmonary embolism	1 (7.14)	1 (7.14)	0	0	0	0
Anemia	1 (7.14)	1 (7.14)	0	0	0	0
Rash maculopapular	1 (7.14)	0	0	1 (7.14)	0	0

### 3.4 Antitumor activity

Although no complete responses (CR) and partial responses (PR) were observed, all patients achieved stable disease, demonstrating the efficacy of this regimen in controlling disease progression. Several patients exhibited prolonged PFS (>10 months), suggesting heterogeneity in antitumor benefit across individuals. Compared with previously reported outcomes of PD-1 monotherapy or Apatinib alone, this study demonstrated a higher DCR and a markedly longer OS.

In the present study, we also explored the potential association between VEGFR-2 expression and clinical outcomes. VEGFR-2 protein expression was assessed by immunohistochemistry (IHC) in tumor samples from 13 patients (As shown in **Table 1**). Due to the limited sample size and the predominance of low or absent VEGFR-2 expression, no statistically significant correlation was observed between VEGFR-2 expression levels and PFS or OS. The single patient with moderate VEGFR-2 expression (++) achieved stable disease lasting more than 10 months, which may suggest a potential trend toward improved disease control, but this observation remains hypothesis-generating and requires validation in larger cohorts.

### 3.5 Other biomarkers

Due to the limited sample size, comprehensive genomic or immunologic analyses were not performed. However, previous studies have suggested that tumor mutational burden (TMB), PD-L1 expression, and alterations in the RTK/RAS pathway may be associated with clinical benefit. Future studies with larger cohorts incorporating histological and molecular profiling are warranted to identify predictive biomarkers and to advance precision medicine in mucosal melanoma.

## 4 Discussion

Mucosal melanoma (MM) represents a rare and aggressive melanoma subtype with a poor prognosis and limited therapeutic options (2, 12). The combination of immune checkpoint inhibitors (ICIs) and anti-angiogenesis agents has emerged as a promising treatment strategy for MM, overcoming the resistance observed with monotherapies, including immune checkpoint inhibitors alone (10, 11, 13–15). In this study, we evaluated the real-world efficacy and safety of SHR-1210, a PD-1 antibody, in combination with Apatinib, a vascular endothelial growth factor (VEGF) receptor inhibitor, as a late-line treatment for patients with BRAF-negative advanced MM.

The results of this study demonstrate promising outcomes with SHR-1210 plus Apatinib in patients with advanced MM. The disease control rate (DCR) reached 100%, with all 13 enrolled patients achieving stable disease (SD). The median overall survival (OS) of 29.9 months (95% CI: 15.26–44.54 months) is noteworthy given the aggressive nature of MM, where five-year survival rates are typically less than 20%. Furthermore, the median progression-free survival (PFS) was 5.17 months (95% CI: 3.27–10.27 months), indicating the ability of this combination to control disease progression in a subset of patients. Although the ORR was limited, due to their good tolerance, no significant depletion of patients’ physical condition, and no serious adverse reactions, patients had the opportunity to receive subsequent treatment.

These findings align with those observed in previous trials combining PD-1 inhibitors with VEGFR inhibitors, such as axitinib, which showed improved progression-free survival and overall survival in patients with metastatic MM (9, 10, 16). For example, a phase Ib trial involving toripalimab and axitinib achieved a response rate of 48.3% with a median PFS of 7.5 months in chemotherapy-naïve patients (16). This supports the hypothesis that targeting both the immune and angiogenic pathways may synergistically enhance antitumor immunity and improve clinical outcomes in patients with MM.

Moreover, the real-world data from this study are consistent with results from other retrospective studies on the combination of anti-PD-1 therapy with anti-angiogenesis agents in advanced solid tumors (17, 18). A study investigating axitinib plus PD-1 inhibitors in the treatment of advanced mucosal melanoma reported a disease control rate of 72.7% and a median time to treatment failure (TTF) of 5.2 months (10). These findings suggest that the addition of VEGF inhibition may help to overcome resistance to immune checkpoint inhibition, making it a potential treatment paradigm for patients with MM.

VEGFR−2 expression was assessed in tumor tissues, with the majority of patients showing low or absent expression; the single patient with moderate expression (++) achieved prolonged stable disease, though no definitive correlation between VEGFR−2 expression and clinical outcomes could be established due to the small sample size. These findings are consistent with the hypothesis that higher VEGFR-2 expression could theoretically enhance the anti-angiogenic effect of Apatinib; however, the present data do not support a definitive predictive role for VEGFR-2 expression in this combination therapy. Future studies with larger sample sizes and standardized IHC protocols are warranted to clarify whether VEGFR-2 expression status can serve as a reliable biomarker for selecting patients with mucosal melanoma who are most likely to benefit from anti-PD-1 plus VEGFR-2 inhibitor combinations.

The safety profile of SHR-1210 plus Apatinib in this study was manageable. The most common treatment-related adverse events (AEs) were mild to moderate and included hand-foot syndrome, fatigue, and hypertension, consistent with those reported for other VEGF inhibitors and PD-1 inhibitors. The occurrence of grade 3 or 4 AEs was relatively low, with only one case of grade 4 abnormal liver function and one case of grade 3 hypertension. Notably, no treatment-related deaths occurred, underscoring the potential for this combination to offer clinical benefits with a manageable toxicity profile. The safety data observed in this study are comparable to those reported in trials investigating axitinib combined with PD-1 inhibitors, where grade 3–4 toxicities were observed in a subset of patients but were manageable through dose adjustments.

These results are consistent with the well-established safety profile of Apatinib and other anti-angiogenic agents. Apatinib, when used in combination with temozolomide in patients with advanced melanoma, has demonstrated a manageable safety profile with most toxicities being grade 1 or 2. These data further support the tolerability of Apatinib in combination with immune checkpoint inhibition, even in heavily pretreated MM patients.

While the sample size of this study was small, it raises the possibility that this combination therapy could be particularly effective in patients who have failed multiple lines of therapy. One of the key strengths of this approach is its potential to address the immunosuppressive tumor microenvironment (TME) that is characteristic of MM. By combining a PD-1 inhibitor like SHR-1210 with a VEGFR inhibitor like Apatinib, the therapy targets both the immune checkpoint pathways and the angiogenesis-driven immune evasion mechanisms in MM, improving T-cell infiltration and enhancing antitumor immunity.

The exploration of biomarkers, such as tumor mutational burden (TMB) and PD-L1 expression, could further refine the treatment strategy for MM. Several studies have suggested that higher TMB and PD-L1 expression are associated with better responses to immune checkpoint inhibitors (19–21). Similarly, alterations in genes involved in the RTK/RAS pathway have been linked to improved response to anti-angiogenic therapies (13, 22). Further investigations are needed to identify predictive biomarkers for MM patients undergoing combination immunotherapy and anti-angiogenesis treatment. Biomarker-driven strategies could allow for better patient stratification and more personalized treatment approaches, ultimately improving clinical outcomes.

Despite these promising results, there are several limitations that should be addressed in future studies. The small sample size and the single-center design of this study limit the generalizability of the findings. Additionally, the absence of a control group prevents definitive conclusions about the comparative efficacy of this combination therapy against other standard treatment regimens for advanced MM. Larger, multicenter randomized trials are needed to validate these results and determine whether SHR-1210 plus Apatinib offers superior efficacy compared to current treatment options.

Moreover, the duration of follow-up in this study was limited, and while the median OS of 29.9 months is encouraging, longer-term data will be needed to assess the durability of response and the potential for long-term survival benefits. The role of Apatinib in the management of MM needs further investigation, particularly in combination with other therapies, to optimize treatment protocols and reduce the burden of toxicities.

## 5 Conclusion

In conclusion, the combination of SHR-1210 plus Apatinib achieved durable disease control with a favorable safety profile in patients with advanced BRAF-negative mucosal melanoma. While objective tumor shrinkage was not observed, the regimen provided clinically meaningful stabilization and prolonged survival. VEGFR-2 expression was assessed in tumor tissues, with the majority of patients showing low or absent expression; the single patient with moderate expression (++) achieved prolonged stable disease, though no definitive correlation between VEGFR-2 expression and clinical outcomes could be established due to the small sample size. These results underscore the importance of integrating anti-angiogenic agents with immunotherapy and lay the groundwork for future biomarker-driven strategies, including larger studies to clarify the predictive role of VEGFR-2 expression, to optimize outcomes in this challenging melanoma subtype.

## Data Availability

The original contributions presented in the study are included in the article/supplementary material. Further inquiries can be directed to the corresponding authors.
